# Molecular Pathology of Human Papilloma Virus-Negative Cervical Cancers

**DOI:** 10.3390/cancers13246351

**Published:** 2021-12-17

**Authors:** Hiroshi Yoshida, Kouya Shiraishi, Tomoyasu Kato

**Affiliations:** 1 Department of Diagnostic Pathology, National Cancer Center Hospital, 5-1-1 Tsukiji, Chuo-ku, Tokyo 104-0045, Japan; 2 Division of Genome Biology, National Cancer Center Research Institute, Tokyo 104-0045, Japan; kshirais@ncc.go.jp; 3 Department of Gynecology, National Cancer Center Hospital, 5-1-1 Tsukiji, Chuo-ku, Tokyo 104-0045, Japan; tokato@ncc.go.jp

**Keywords:** cervical cancer, human papillomavirus (HPV), HPV-independent cancer, pathology, genetics

## Abstract

**Simple Summary:**

Although cervical cancer is mainly caused by infection with human papillomavirus (HPV), some cervical cancers test negative for HPV. As these HPV-negative cervical cancers are often diagnosed at an advanced stage and have a poor prognosis, it is important to understand their molecular pathology. Here, we provide an overview of the potential causes of false-negative HPV tests, as well as the histology, genetic profile, and pathogenesis of HPV-negative cancers.

**Abstract:**

Cervical cancer is the fourth most common cancer in women worldwide and is predominantly caused by infection with human papillomavirus (HPV). However, a small subset of cervical cancers tests negative for HPV, including true HPV-independent cancers and false-negative cases. True HPV-negative cancers appear to be more prevalent in certain pathological adenocarcinoma subtypes, such as gastric- and clear-cell-type adenocarcinomas. Moreover, HPV-negative cervical cancers have proven to be a biologically distinct tumor subset that follows a different pathogenetic pathway to HPV-associated cervical cancers. HPV-negative cervical cancers are often diagnosed at an advanced stage with a poor prognosis and are expected to persist in the post-HPV vaccination era; therefore, it is important to understand HPV-negative cancers. In this review, we provide a concise overview of the molecular pathology of HPV-negative cervical cancers, with a focus on their definitions, the potential causes of false-negative HPV tests, and the histology, genetic profiles, and pathogenesis of HPV-negative cancers.

## 1. Introduction

### 1.1. Human Papillomavirus and Cervical Cancer

Cervical cancer is the fourth most common cancer in women worldwide, accounting for approximately 6.5% of all female cancer cases [1]. The leading cause of cervical cancer is considered to be a persistent infection with human papillomavirus (HPV), particularly high-risk HPVs. HPV has a double-stranded DNA genome of up to 8 kbp that encodes six early genes (E1, E2, E4, E5, E6, and E7) and two late genes (L1 and L2) that constitute the viral capsid [2]. During the early stages of infection, HPV DNA replicates from free DNA in the basal cells at the cervix and integrates into the host genome as the infection progresses, thereby upregulating the E6 and E7 oncogenes [3]. 

HPVs are classified by genotype according to differences in their L1 DNA sequence of >10% compared to other established genotypes, numbered by their date of discovery [4]. Although more than 200 HPV genotypes have been identified [5], a small subset is known to be carcinogenic or probably carcinogenic: HPV-16, 18, 31, 33, 35, 39, 45, 51, 52, 56, 58, 59, and 68 [6]. Approximately 90% of cervical carcinomas are caused by high-risk HPV, with HPV-16 accounting for 50–60% and HPV-18 for 10–20%, and the remaining 10% caused by other oncogenic types [7,8]. Several additional HPV genotypes are possibly carcinogenic or low-risk; for example, HPV-6 and 11 are low-risk types that are prevalent in benign condyloma acuminatum. 

Nearly all squamous cell carcinomas (SqCCs) of the cervix and most pre-invasive lesions, including high-grade squamous intraepithelial lesions (HSILs) (previous terminology: grade 2–3 cervical intraepithelial neoplasia; CIN2-3) contain HPV. Although the increased global availability of HPV vaccines has decreased the number of deaths from HPV-positive cervical cancer [9,10] and the sensitivity of HPV testing has significantly increased in recent years, a small but significant percentage of cervical carcinomas remain HPV-negative. Indeed, true HPV-negative cervical cancer is expected to persist in the post-vaccination era and is frequently diagnosed at an advanced International Federation of Gynecology and Obstetrics (FIGO) stage with a poor prognosis [11,12]. Therefore, it is important to understand the molecular pathology of HPV-negative cervical cancer to develop appropriate patient management strategies.

### 1.2. Definition of HPV-Independence in Cervical Cancer

There is currently no consensus definition of HPV-negative cervical carcinoma; however, we believe that the term should be reserved for confirmed primary cervical cancer cases without a plausible explanation for a false-negative HPV test result. There are several HPV tests with different sensitivities and specificities [13], including nucleic acid signal amplification methods such as polymerase chain reaction (PCR; e.g., Cobas^TM^ HPV and BD’s Onclarity^TM^ HPV) and transcription-mediated amplification (e.g., APTIMA^TM^ HPV), and non-nucleic acid amplification methods such as hybridization capture (e.g., HC2^TM^) and invader chemistry (e.g., Cervista^TM^ HPV) [14]. HPV in situ hybridization [15] in formalin-fixed paraffin-embedded sections has also been used in surgical pathology [16]. The causes of false-negative HPV test results and their associated diagnostic pitfalls are summarized in Table 1. 

HPV-negative cervical cancers account for up to 11% of cervical cancers [17,18,19,20]; however, a meta-analysis of >30,000 women with invasive cervical cancer exhibited a gradual decline in the rate of HPV-negativity [21], from 14.1% in 1990–1999 to just 7.1% in 2006–2010. This decline could be attributed to the increased sensitivity of HPV testing and accuracy of diagnosing non-cervical cancer. A recent study of the Cancer Genome Atlas (TCGA) revealed that ~5% of primary cervical cancers are HPV-negative [22], while several less common pathological subtypes are consistently HPV-negative [23,24] (Table 2). Notably, the geographic distribution of HPV-negative cancer may vary. For example, in a study of Belgian women diagnosed with cervical cancer prior to 2000, 13% of them had HPV-negative disease [25], while another study reported 7.1% of women with HPV-negative cervical cancer between 2001 and 2008 [26]. The percentage of global HPV-negative cervical cancer cases ranges from 7% to 11% [17,18,19,20]. The HPV-negative rate was reduced to 5% in TCGA studies with samples that were primarily collected in the United States. However, we should consider several possible false-negative HPV test result scenarios before diagnosing HPV-negative cervical cancer:

1. Deletion of HPV DNA fragments targeted by HPV testing during host genome integration [27,28,29,30];
2. Very low viral load in latent HPV infections [8];
3. Cervical cancer caused by non-high-risk HPV that is undetectable using HPV testing [20,31,32,33]; and
4. Inadequate sampling and various pre-analytical factors associated with HPV testing [30,34,35].

An extensive review of HPV testing and the factors that affect false-negative results is beyond the scope of this review and has been described thoroughly in previous publications [35,36,37]. Nevertheless, these possible causes of false-negative HPV testing should be considered when diagnosing HPV-negative cervical cancers. Non-cervical cancers misinterpreted as primary cervical cancer can include the cervical involvement of endometrial carcinoma or distant metastases from other primary HPV-negative tumors. Indeed, Petry et al. reported that approximately two-thirds of HPV-negative cervical carcinomas were not a cervical primary [20], while Hopenhayn et al. reported close morphological similarity between a significant proportion of HPV-negative cervical adenocarcinomas and endometrial carcinomas [38]. In addition, 3.7% of female genital metastatic tumors have been reported to involve the uterine cervix [39]. The primary sites of metastatic tumors in order of frequency include the ovaries, colorectum, stomach, breast, kidneys, lungs, pancreaticobiliary tract, fallopian tubes, appendiceal carcinoids, mesotheliomas, and melanomas [39,40]. 

**Table 2:** HPV infection rates in different histological types of cervical cancer.

Histological Type	HPV Positivity (%)	References
Squamous cell carcinoma	87–100	[8]
Usual type endocervical adenocarcinoma	72–100	[34,41,42]
Mucinous adenocarcinoma	83–100	[42]
Gastric-type adenocarcinoma	0	[41]
Clear cell adenocarcinoma	0–28	[34,41,43]
Mesonephric adenocarcinoma	0	[42]
Endometrioid adenocarcinoma	0–27	[34,41,42]
Serous adenocarcinoma	0–30	[34,41,44]
Adenosquamous carcinoma	81–86	[8,34,41]
Adenoid basal carcinoma	80–100	[45,46]
Carcinosarcoma	100	[47]
Neuroendocrine carcinoma	86–100	[48,49]

### 1.3. WHO Histopathological Classification of Cervical Cancers Based on HPV Infection Status

**Figure 1:**
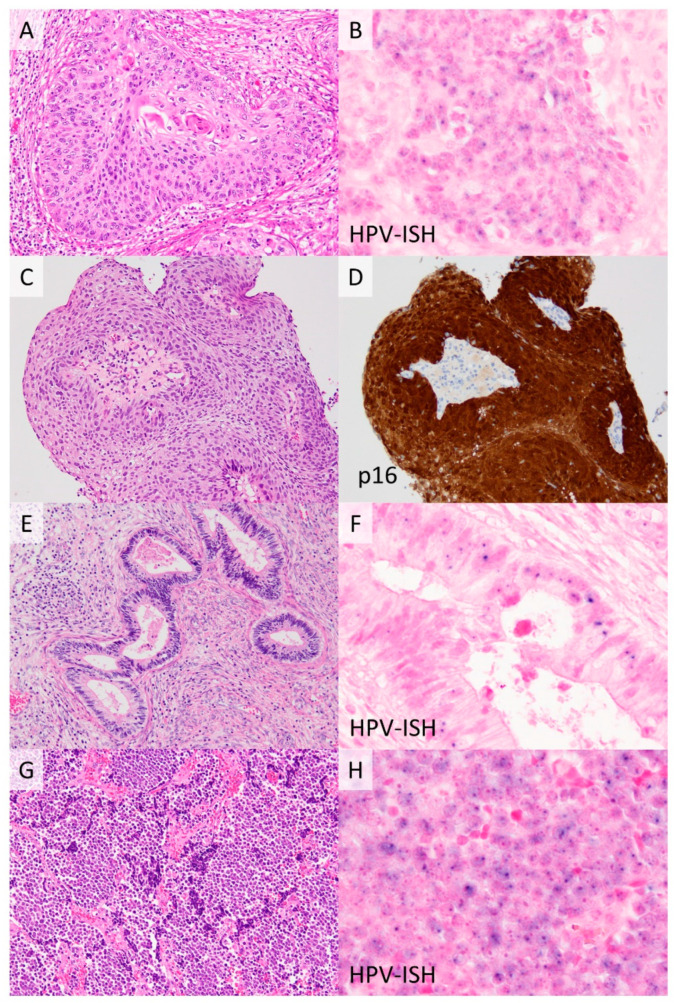
Representative histological types of human papillomavirus (HPV)-associated cervical cancers. Squamous cell carcinoma (**A**) HPV in situ hybridization reveals small blue dot signals in tumor cells (**B**). Squamous cell carcinoma showing a so-called block positive pattern of p16 immunohistochemistry (**C**,**D**). Usual-type endocervical adenocarcinoma (**E**) shows HPV-positivity (**F**). Small cell neuroendocrine carcinoma (**G**) is reportedly HPV positive (**H**) in most cases. ((**A**,**C**,**E**,**G**); **H**&**E** stain, ×200, (**D**), ×200, (**B**,**F**,**H**), ×1000).

The most recent WHO classification of female genital tumors (2020, 5th ed.) divides cervical carcinomas and their precursors based on their association with HPV infection [50] (Table 3). Squamous cell carcinoma (SqCC) and adenocarcinoma, which are the two most common tumor types, are classified as either HPV-associated or HPV-independent (Figure 1). Although the majority of SqCCs are HPV-associated, some rare cases of HPV-independent SqCC have been described [50]. The WHO acknowledges that it is impossible to distinguish between HPV-associated and -independent SqCC using morphological methods, instead of requiring p16 immunohistochemistry (IHC) or HPV testing. Since current treatment strategies do not rely on HPV independence [51,52] it is not necessary to define these categories when ancillary techniques are unavailable. 

**Figure 2:**
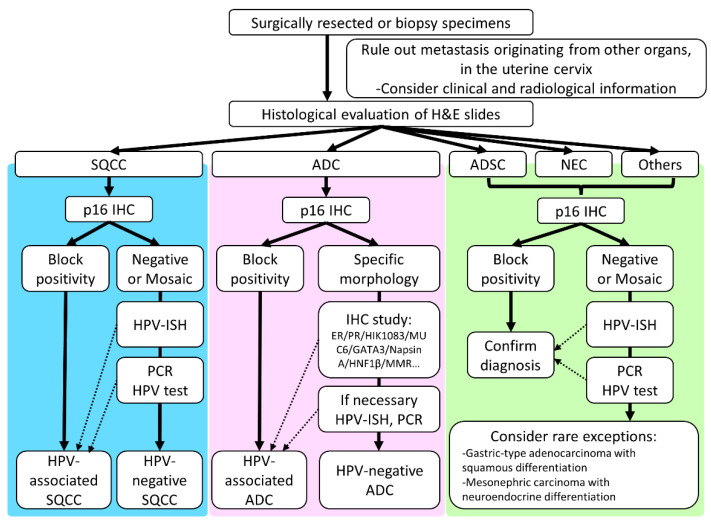
Histological typing of invasive cervical cancer. A thorough examination of clinical and radiological data, as well as H&E-stained slides, is essential. Ancillary tests such as p16 immunohistochemistry, HPV in situ hybridization, or PCR-based HPV testing should be used for accurate histological typing. Abbreviations: SQCC, squamous cell carcinoma; ADC, adenocarcinoma; ADSC, adenosquamous carcinoma; H&E, hematoxylin and eosin staining; HPV, human papillomavirus; IHC, immunohistochemistry; ISH, in situ hybridization; NEC, neuroendocrine carcinoma; PCR, polymerase chain reaction.

Unlike SqCC, HPV-associated adenocarcinomas can be distinguished morphologically by the presence of conspicuous and identifiable apical mitoses and karyorrhexis at low power magnification (Figure 1). These include glands with smooth luminal surfaces and pseudostratified columnar epithelial cells with enlarged, elongated, and hyperchromatic nuclei in well-to-moderately differentiated glands. Although p16 IHC block positivity is useful (Figure 1), it can be strongly or diffusely positive in some HPV-independent adenocarcinomas. HPV-associated adenocarcinomas are classified into two histological types: usual and mucinous. Usual type HPV-associated adenocarcinoma is the most common, accounting for ~75% of cases and including those with mucin secretion in 0–50% of cells and a villoglandular architecture. Mucinous HPV-associated adenocarcinoma is subtyped into mucinous, not otherwise specified (NOS) adenocarcinoma, intestinal adenocarcinoma (goblet or enteroendocrine cell differentiation in 50% of the tumor), signet-ring cell adenocarcinoma (non-cohesive cells with a signet-ring morphology and cytoplasmic mucin), and invasive stratified mucin-producing carcinoma [50]. Conversely, HPV-independent adenocarcinomas include gastric, clear cell, mesonephric, endometrioid, and serous carcinomas. These changes in classification reflect the International Endocervical Adenocarcinoma Criteria and Classification (IECC) 2018 recommendation [24]. Other tumors including neuroendocrine, lymphoid, mesenchymal, melanocytic, and metastatic tumors are described separately in other chapters, alongside tumors from other female genital tracts [50]. Here, we describe the molecular pathological features of different cervical cancers based on the 2020 WHO classification. Figure 2 illustrates a flowchart that describes a practical histological typing method for invasive cervical cancer.

## 2. Pathology and Genetics of HPV-Independent Cervical Cancer

### 2.1. HPV-Independent SqCC

HPV-independent SqCC of the uterine cervix is defined by the 2020 WHO classification as an HPV-independent squamous tumor with stromal invasion and/or exophytic invasion [50]. Even with the most sensitive HPV detection methods, ~5–7% of all cervical SqCC cases are HPV negative, and the clinical, macroscopic, and microscopic features are indistinguishable from those of HPV-associated SqCC [11,56]. SqCC can be polypoid or deeply infiltrative, with various histological patterns, including keratinizing, non-keratinizing, papillary, basaloid, and verrucous types. Thus, negative p16 IHC with a suitable positive internal control is required to diagnose HPV-independent SqCC, and HPV molecular testing is recommended [50]. Notably, HPV-independent SqCC is often diagnosed at an advanced stage, resulting in a lower survival rate [11,56,57,58,59,60]. 

Considerable data has been gathered from the genomic analysis of HPV-positive cervical carcinoma. Whole exome sequencing of four large cohorts, including TCGA (*n* = >100) [22,61,62,63], identified numerous significant driver mutations (Table 4): *PIK3CA* (17–26%), *FBXW7* (11–13%), *EP300* (6–11%), *HLA-A* (8%), *PTEN* (8%), *ARID1A* (7%), *NFE2L2* (7%), *KRAS* (6%), *ERBB3* (6%), *HLA-B* (6%), and *MAPK1* (5%) [22,61,63]. Moreover, driver copy number alterations have been found in chromosome 3q gain (66%), *YAP1* (16%), *CD274* (8%), *PTEN* (8%), and *BCAR4* (7%) [22]. Another study detected an apolipoprotein B editing complex (APOBEC) signature in 78% of exomes [64] associated with various driver mutations, suggesting that APOBEC mutagenesis plays major role in cervical cancer carcinogenesis [22]. 

Conversely, genetic studies of HPV-independent SqCC are scarce and include a TCGA study of just four SqCC cases without HPV infection and two with an “endometrial-like” mutational profile, including *PTEN* gene alterations [22] (Table 5). Although the genetic alterations in HPV-negative SqCC remain largely unexplored, the “endometrial-like” mutational profile of HPV-independent SqCC seems similar to that of endometrial endometrioid carcinoma [67], which might reflect its pathogenesis. Endometrial SqCC is recognized as an endometrioid carcinoma with overwhelming squamous differentiation [68]. One possible explanation for HPV-negative SqCC is endometrial SqCC arising in the lower uterine segments and invading the uterine cervix. No definite precursor (such as HSIL) has been identified for HPV-independent SqCC; however, true HPV-independent SqCC is well-established in vulvar cancer and reportedly develops from differentiated vulval intraepithelial neoplasia precursor lesions, which are often associated with lichen sclerosus or planus [69,70,71]. Somatic *TP53* mutations have been detected in ~80% of HPV-independent vulvar SqCC, and activating mutations in *NOTCH1/2*, *HRAS*, and *PIK3CA* are also common [71,72]. Given the absence of established precursor lesions for HPV-independent cervical SqCC and their mutation profiles, a comparable type of HPV-independent cervical SqCC is exceptionally rare. However, more clinical evidence and molecular analysis are needed to elucidate the molecular abnormalities of HPV-negative squamous cell carcinoma. Most recently, Ruiz and colleagues reported genomic and transcriptome tumor profiles and patient survival outcomes of cervical cancer with undetectable HPV. Although detailed information of histological type was not provided, patients with HPV-negative cervical cancer had worse progression-free and overall survival outcomes compared to HPV-positive patients. Furthermore, TP53, ARID1A, PTEN, ARID5B, CTNNB1, CTCF, and CCND1 were identified as significantly mutated genes (SMGs) enriched in HPV-negative cancers, with converging functional roles in cell cycle progression. Notably, they identify palbociclib as a potential treatment strategy for patients with HPV-negative cervical cancer [73].

### 2.2. Gastric-Type Adenocarcinoma

**Figure 3:**
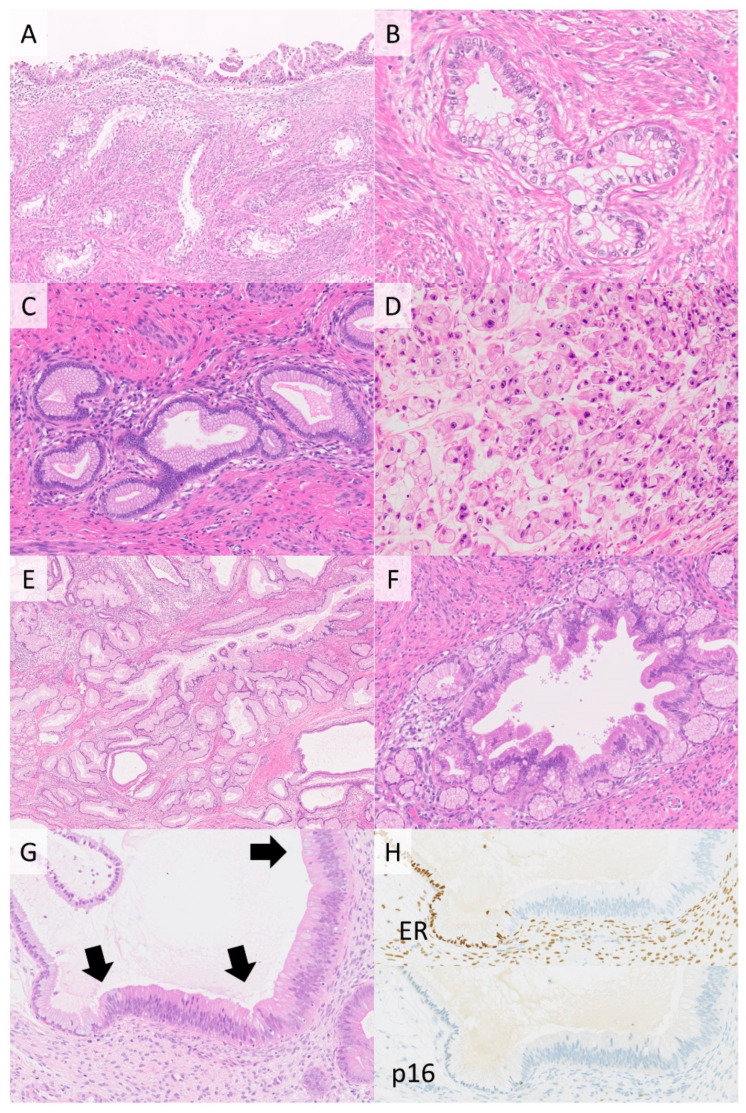
Gastric-type adenocarcinoma (GAS) of the cervix. GAS is a representative histological type of HPV-independent cervical cancers. Irregular glands (**A**) are composed of tumor cells with clear to pale eosinophilic cytoplasm and enlarged nuclei (**B**). GAS shows histological heterogeneity: very well-differentiated adenocarcinoma so-called “adenoma malignum”(**C**) whereas poorly differentiated adenocarcinoma (**D**). Some GASs have adjacent lobular endocervical glandular hyperplasia (LEGH) (**E**) and atypical LEGH is postulated as one of the pre-invasive lesions of GAS (**F**). Gastric-type adenocarcinoma in situ ((**G**), black arrows) shows negativity of immunohistochemistry for estrogen receptor and p16 (**H**). ((**A**,**E**) **H**&**E** stain, ×100; (**B**–**D**,**F**,**G**), **H**&**E** stain, ×200; (**H**) ×200).

Gastric-type adenocarcinoma (GAS) of the cervix, first described in the 1990s, is the second most common type of cervical adenocarcinoma [83] and is an established entity with distinct histological characteristics, IHC profiles, and clinical behaviors [50]. Until now, this entity has included a wide morphological spectrum, from highly differentiated “adenoma malignum” to poorly differentiated carcinoma [84] (Figure 3). Studies have repeatedly confirmed that GAS is HPV-negative using PCR and in situ hybridization [85,86]. Since GAS is more aggressive than HPV-associated endocervical adenocarcinoma and frequently presents with more advanced disease [87,88], it shows poor clinical outcomes, even at stage I [89], and presents with an unusual spread [24,89] and chemoresistance [90]. 

Microscopically, gastric differentiation is described as tumor cells with distinct cell borders and voluminous clear to pale eosinophilic cytoplasm (Figure 3) [91] and may resemble adenocarcinoma cells of gastric and pancreatobiliary origin. Morphological intratumor heterogeneity is a well-recognized feature of GAS, with the same tumor having well-differentiated, minimally deviated adenocarcinoma (MDA)-like components adjacent to poorly differentiated carcinoma. GAS cells contain acidic mucin and express similar immunomarkers to gastric mucus cells, such as HIK1083, lysozyme, and pepsinogen II [87]. In these tumors, p16 staining is usually focal or negative [85,87,92], while some cases of usual-type adenocarcinoma reportedly present mixed features of GAS and usual-type endocervical adenocarcinoma [24,85,93,94].

Recent evidence has revealed the molecular profile of GAS (Table 5) [74,75,76,78]. For instance, Garg et al. described genetic changes in 161 cancer driver genes in 14 GAS cases using next-generation sequencing, identifying gene alterations in *TP53 (50%), MSH6* (43%), *CDKN2A/B* (36%), *POLE* (36%), *SLX4* (36%), *ARID1A* (29%), *STK11* (29%), *BRCA2* (21%), and *MSH2* (21%) [77]. The study also discovered mutations in pathways related to DNA damage repair, the cell cycle, Fanconi anemia, and PI3K-AKT signaling. According to Hodgeson et al., *TP53* (46%), *KRAS* (36%), and *PIK3CA* (18%) were the most often altered genes in 11 GAS cases [78], whereas Lu et al. found that *TP53* (53%), *STK11* (33%)*, CDKN2A* (27%)*, ARID1A* (20%), and *PTEN* (20%) were the most frequently mutated genes in 15 cases of GAS, with frequent *ERBB2* amplification (13%) [76], and primarily affected the cell cycle and PI3K/AKT signaling pathways. Park et al. discovered altered *TP53* (52.4%), *STK11*, *HLA-B*, *PTPRS* (19%), and *FGFR4* (14.3%) expression in 21 cases of GAS [75] with genes involved in signal transduction, DNA damage repair, and epithelial-mesenchymal transition. Recently, Selenica et al. examined 68 cases of GAS and discovered that most somatic mutations occurred in *TP53* (41%), *CDKN2A* (18%), *KRAS* (18%), and *STK11* (10%), with potentially targetable mutations identified in *ERBB3* (10%), *ERBB2* (8%), and *BRAF* (4%) [74]. In addition, these studies revealed that GAS has more *TP53, STK11, CDKN2A, ATM*, and *NTRK3* [78] mutations and fewer *PIK3CA* mutations [74] than typical type endocervical adenocarcinoma. MDA, a very well-differentiated form of GAS, is part of the tumor spectrum and is known to be associated with Peutz-Jeghers syndrome characterized by germline mutations in *STK11* [95]. Indeed, somatic *STK11* mutations were confirmed in 55% of 11 mucinous MDAs and 5% of 19 mucinous adenocarcinomas [96]. 

Although the genetic alterations in GAS remain unclear, precursor lesions have been investigated. Widely accepted benign lesions include simple gastric metaplasia and lobular endocervical glandular hyperplasia (LEGH) [97]. Postulated premalignant lesions include atypical LEGH [98] and related lesions referred to as “gastric-type adenocarcinoma in situ (gAIS)” [99,100,101] (Figure 3). It is particularly important to elucidate the molecular profile of gAIS for early detection as screening is not effective for this clinically aggressive HPV-independent adenocarcinoma.

### 2.3. Clear Cell Carcinoma

**Figure 4:**
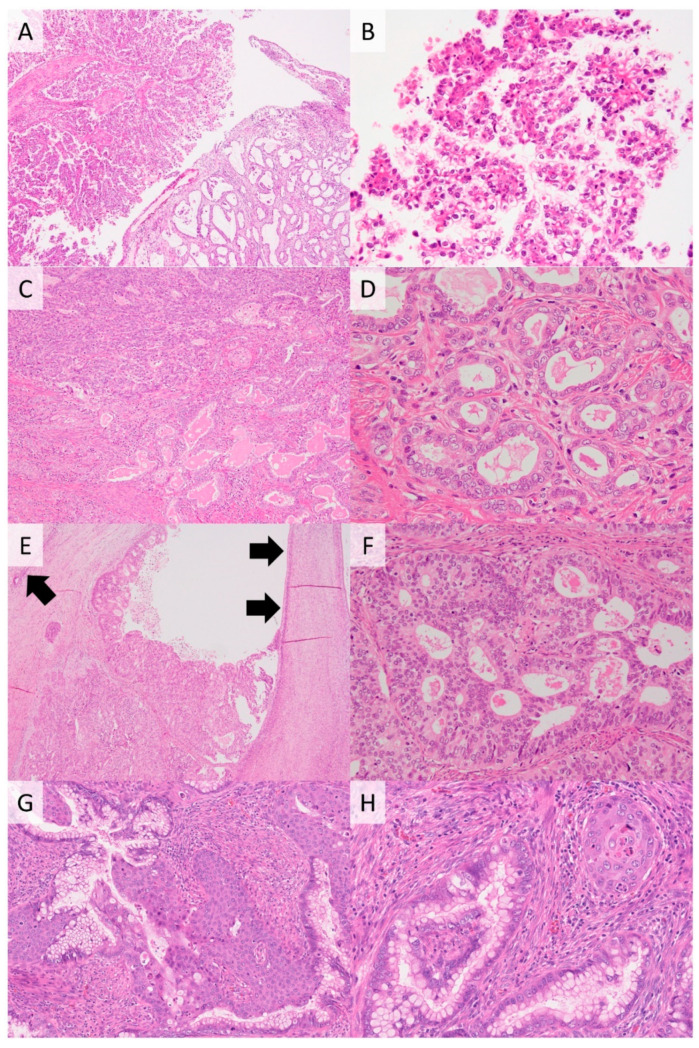
Other types of HPV-independent cervical cancers. Clear cell carcinoma showing papillary structure (upper left) and tubulocystic pattern (lower right) (**A**). Tumor cells have clear cytoplasm and enlarged nuclei with prominent nucleoli (**B**). Mesonephric carcinoma showing various histological patterns such as corded and spindled (upper left) and tubular pattern (lower right) (**C**). Cuboidal tumor cells with pale to eosinophilic cytoplasm forming glands with eosinophilic intraluminal secretions (**D**). Cervical endometrioid adenocarcinoma arising in endometriosis ((**E**), black arrows). Columnar tumor cells showing cribriform glands (**F**). Gastric-type adenocarcinoma showing prominent squamous differentiation (**G**) and this tumor is an HPV-independent adenosquamous carcinoma (**H**). ((**A**,**C**,**E**,**G**), **H**&**E** stain, ×100; (**B**,**D**,**F**,**H**), **H**&**E** stain, ×200).

Clear cell carcinoma (CCC) is primarily composed of clear or hobnail cells in solid, tubulo-cystic, or papillary architectural patterns (Figure 4A,B) [85], and nuclei with identifiable high-grade characteristics such as hyperchromasia, pleomorphism, and prominent nucleoli [24]. Typically, CCC tumors contain an abundance of glycogen-rich cytoplasm and, occasionally, hyaline globules. Uncommon malignancies such as vaginal CCC have been linked to in utero exposure to diethylstilbestrol (DES), a synthetic estrogen administered during the 1948–1970s to prevent miscarriage, premature labor, and related complications [102]. The age distribution of cervical CCC is bimodal (young adults vs. postmenopausal women). The risk of disease in DES-exposed patients peaks at 19 years of age and remains throughout later life, with the ectocervix and anterior upper part of the vagina is the most frequent site of involvement. In non-DES-exposed patients, the peak age ranges from pediatric to postmenopausal and the endocervix is the primary site of involvement. These tumors test IHC-positive for PAX8, HNF1β, and napsin-A, while p53 and p16 can be an abnormal pattern or not, and ER and PR are usually negative. However, these tumors consistently display CEA negativity, suggesting that it could be used to differentiate CCC from GAS with voluminous clear cytoplasm. 

Unfortunately, the molecular basis of cervical CCC is poorly understood. Boyd et al. [103] found microsatellite instability in 100% of DES-exposed cases and 50% of non-DES-exposed cases, but no mutations in *KRAS*, *HRAS*, *WT1*, *ER*, or *TP53*. Recently, Lee et al. reported a case of a woman exposed to DES in utero who developed cervical CCC with a somatic mutation in *POLE* [79]. Mills et al. assessed several cases of endocervical adenocarcinoma, including one with CCC with MMR deficiency but without MMR protein loss or Lynch syndrome association [104]. Conversely, Nakamura et al. described a patient with Lynch syndrome who developed synchronous cervical CCC with the loss of MSH2 and MSH6 [105]. Another study used IHC to examine molecular pathways and discovered that some cervical CCC cases lack PTEN but test positive for pAKT and mammalian target of rapamycin (mTOR), with one case demonstrating *HER2* amplification [43]. Most recently, Jenkins et al. reported one *TP53* and one *PIK3CA* mutation in three HPV-negative cervical CCCs [44]. Although the pathogenesis and precursor lesions of cervical CCC remain unknown, the reported genetic alterations suggest that some non-DES-exposed CCC cases may be derived from cervical endometriosis or endometrial metaplasia similar to ovarian CCC or endometrioid carcinoma arising from endometriosis [106]. Although unconfirmed by genetic analysis, Talia et al. reported tubo-endometrial metaplasia that was proximal to cervical CCCs as a possible precursor of CCC [53].

### 2.4. Mesonephric Carcinoma

Mesonephric carcinomas are malignant neoplasms with mesonephric differentiation [50] that are not associated with high-risk HPV infection [107] but can be clinically aggressive and metastasize to distant organs [108]. Mesonephric carcinomas typically develop in the lateral-to-posterior cervical wall and can be deeply invasive, bulky, or exophytic. These tumors have a heterogeneous architecture, with glandular, tubular, solid, papillary, retiform, sex cord-like, and spindled/sarcomatoid growth patterns (Figure 4C,D). Dense eosinophilic secretions, similar to those seen in benign mesonephric remnants, are observed within the glandular luminal spaces [50,109], and mesonephric carcinomas can be positive for cytokeratin and epithelial membrane antigens (e.g., calretinin, CD10, and vimentin). Mesonephric carcinomas are typically negative for estrogen and progesterone receptors, as well as CEA, but may express PAX8 and, occasionally p16 [85]. Because mesonephric carcinomas are not caused by HPV infection, block positivity of p16 immunostaining is not usually observed. 

Accumulating evidence of molecular profile of mesonephric carcinomas revealed canonical *KRAS* mutations in most tumors (81%) and some activating *NRAS* mutations (Table 5) [80]. *ARID1A/B*, *SMARCA4,* and chromatin-remodeling gene mutations were also common (62%), and one-third of patients harbored *BCOR/BCORL1* mutations. No cases had *PIK3CA* or *PTEN* alterations, unlike usual-type adenocarcinoma, and none had microsatellite instability; however, some chromosomal structural abnormalities have been observed in mesonephric carcinomas, including copy number gains in 1q, the loss of 1p, and the gain of chromosomes 10 and 12 [80]. Montalvo et al. also reported a case of mesonephric carcinoma with genomic alterations in *KRAS* and *CTNNB1*, the gain of chromosome 1q, a low tumor mutation burden, and the absence of microsatellite instability [81]. 

Although mesonephric carcinoma precursor lesions have not yet been fully characterized, no activating *KRAS* or *NRAS* mutations were found in a follow-up study of ten mesonephric hyperplasia cases [110]. Notably, Kim et al. reported two atypical cases of mesonephric hyperplasia with co-existing mesonephric carcinoma harboring the same *KRAS* mutations, one of which had chromosome 1q gain. Thus, atypical mesonephric hyperplasia could be a pre-invasive lesion in which *KRAS* mutation and chromosome 1q gain may contribute to the tumorigenesis process of mesonephric carcinoma [54]. Recently, a case of cervical mesonephric adenocarcinoma with *FGFR2* mutation was reported [111]. 

### 2.5. Endometrioid Carcinoma

Primary cervical endometrioid carcinoma arises from the cervix and exhibits endometrioid morphologic characteristics, such as tumor cells lacking mucin with a sparse, eosinophilic cytoplasm resembling endometrial epithelium. These tumors are rare, constituting less than 5% of all cervical adenocarcinomas [50], and do not appear to be associated with high-risk HPV infection [112]. Prior to the 2020 WHO classification, the IECC proposed [24] a more precise definition of endometrial adenocarcinoma to improve inter-observer reproducibility among pathologists. This system relied on “confirmatory endometrioid diagnostic features”, including focally identified low-grade endometrioid glands lined by columnar cells, pseudostratified nuclei with mild atypia, squamous differentiation, and/or the presence of endometriosis (Figure 4E,F). Endometrioid carcinomas display no morphological characteristics of HPV-associated adenocarcinomas, such as conspicuous and identifiable apical mitoses and karyorrhexis at low power magnification. Indeed, a large proportion of tumors in one study would have been diagnosed with endometrioid adenocarcinoma using the 2014 WHO criteria [113], yet only 1.1% (3/371) of cases fell into this category when strict HPV-related criteria were applied, with HPV-negativity confirmed using ISH. Endometrioid carcinoma reportedly develops from cervical endometriosis [55,114], and more common scenarios such as endometrial endometrioid adenocarcinoma extending into the cervix or unusual presentations of usual type endocervical adenocarcinoma should be considered before diagnosing cervical endometrioid adenocarcinoma. Although the characteristic genetic abnormalities in cervical endometrioid adenocarcinoma remain unknown, Jenkins et al. recently reported eight HPV-negative cases of cervical endometrioid adenocarcinoma harboring various somatic gene mutations, including *PIK3CA* (50%), *PTEN* (50%), *CTNNB1* (3/8%), *FBXW7* (25%), *KRAS* (1/8%), *AKT1* (1/8%), and MSI-H (1/8 %) [44]. Therefore, it would be interesting to determine whether endometrial carcinoma has molecular subtypes similar to endometrial or ovarian endometrioid carcinoma [115,116]. Molecular similarities between endometriosis-related neoplasms are expected, but this has not yet been thoroughly investigated. In any case, the discovery of therapeutic targets such as MSI-H under the aforementioned conditions would be clinically relevant.

### 2.6. Serous Carcinoma

Primary cervical serous carcinoma is exceptionally rare and often has a papillary architecture with prominent tufted papillae lined by tumor cells with high-grade nucleus atypia [85]. However, its existence is now questioned on the basis of two areas of ambiguity [117]. Firstly, “serous carcinoma” is a morphological variation in HPV-positive usual-type endocervical adenocarcinoma [118]. Secondly, “serous carcinoma” can be a drop metastasis of a high-grade serous carcinoma (HGSC) of the ovary, fallopian tube, or endometrial serous carcinoma misdiagnosed as cervical primary carcinoma. Therefore, cervical serous carcinoma is suspected to be a mixture of two different tumors based on past observations of clinicopathological features. The age distribution of patients with serous carcinoma is bimodal [119,120], with pre- and post-menopausal cases exhibiting distinct clinicopathological characteristics. Premenopausal patients are more likely to be HPV positive [34], contain usual-type endocervical adenocarcinoma components, and demonstrate WT-1 negative IHC staining. Conversely, postmenopausal cases are more likely to be HPV negative, contain only serous carcinoma components, and demonstrate WT-1 positivity [112]. In addition, a thorough examination of the fallopian tubes of postmenopausal cases revealed serous tubal intraepithelial carcinomas (STICs) with *TP53* mutations shared with cervical tumors [121]. Collectively, most premenopausal cases are thought to be morphological variants of the common endocervical adenocarcinoma, whereas postmenopausal cases may exhibit drop metastases of HGSC of the fallopian tubes and ovaries. The origin of genuine serous carcinoma of the cervix, which was diagnosed after ruling out these possibilities, is unknown.

To date, no studies have reported the genetic features of HPV-negative serous carcinoma of the cervix, thereby ruling out these two possible differential diagnoses. However, Jenkins et al. recently reported that cervical serous carcinoma (*n* = 6, HPV-negative) harbors mutations in *TP53* (50%), *KRAS* (33%), *PIK3CA* (17%), and *PTEN* (17%) [44]. The possibility of drop metastasis in endometrial serous and adnexal HGSC should always be considered since tumor cells are detected in ~40% of uterine specimens in advanced HGSC [122]. Therefore, a diagnosis of cervical serous carcinoma should only be made after thorough and extensive sampling of the endometrium and fallopian tubes using the sectioning of the fimbriated end of the fallopian tube (SEE-FIM) technique. Indeed, recognizing the correct primary tumor can allow the identification of effective therapeutic targets to benefit the patients. For instance, approximately 30% of patients with endometrial serous carcinoma are HER2-positive, making trastuzumab-containing regimens more effective [123], whereas approximately half of the pelvic HGSCs are positive for homologous recombination repair deficiency (HRD) [124] and can be treated using PARP inhibitors [125].

### 2.7. HPV-Independent Adenosquamous Carcinoma

Cervical adenosquamous carcinoma (ASC) is defined as a malignant epithelial tumor that exhibits both squamous and glandular differentiation [50] and reportedly accounts for 5–6% of cervical cancers [126,127]. The 2020 WHO definition of ASC does not include an HPV-independent classification and ASC is primarily thought to be an HPV-related tumor, most commonly HPV-16 and -18 [50]. HPV is detected in up to 86% of ASCs [41,127]; however, the genetic alterations in ASC have not yet been elucidated, and even a TCGA study of only three ASC cases included just one HPV-negative case [22]. We previously evaluated 14 ASC samples using targeted DNA sequencing and identified genetic alterations in *PIK3CA* (2/14%), *TP53* (1/14%), and *KRAS* (1/14%), *HER2* amplification, and *PTEN* copy number loss [49], which partly resembles the genetic profile of HPV-positive SqCC or adenocarcinoma. Although no recurrent gene alterations were identified in the three HPV-negative ASC cases, true HPV-independent ASCs may exist as a result of squamous differentiation in HPV-independent cancers, such as squamous differentiation in endometrioid carcinoma derived from cervical endometriosis. Recently, we reported two GAS cases with squamous differentiation and HPV-negative ASC formation [128]. Both had an advanced stage (pT2bN1) with predominant GAS and merged SqCC components without p16-block positivity or HPV DNA. Gastric-type AIS was confirmed in both cases (Figure 4G,H). These tumors are expected to have genetic abnormalities similar to the original HPV-negative adenocarcinoma, and we intend to investigate their genetic profiles in the future. 

### 2.8. HPV-Independent Neuroendocrine Carcinoma

Neuroendocrine carcinomas (NECs) of the cervix are invasive carcinomas that display neuroendocrine differentiation and account for less than 2% of all cervical cancers. However, these tumors are aggressive and have a high tendency for lymph node metastases and spread to distant organs [129], with HPV detected in 85–100% of cases [48,49,130]. Alejo et al. found that 86% of NECs (*n* = 49) had HPV DNA, while 55% harbored HPV16, 41% had HPV18, and 4% were positive for other HPV types [130]. Previously, we identified HPV in all 25 cases of cervical NEC, with HPV-18 being the most common [49]. Although the clinicopathological characteristics of HPV-independent NEC remain unknown, most NECs are thought to be the result of trans-differentiation from SqCC or adenocarcinoma [131], with HPV-positive SqCC in situ or usual-type adenocarcinoma in situ being common precursor lesions. There have been some reports of HPV-negative NECs, implying that these tumors are a mix of false-negative and true HPV-negative cases. Eskandar et al. reported that 14% (14/97) of NEC samples in their study were HPV-negative [48] and revealed a significantly higher frequency of several gene alterations in HPV-negative NECs including *PIK3CA* (17% vs. 36%), *TP53* (11% vs. 43%), *PTEN* (8% vs. 50%), *ARID1A* (5% vs. 36%), and *RB1* (4% vs. 36%). Although there was no difference in the frequency of *TMB-H* between HPV-positive and HPV-negative NECs, only *HPV-16* and *18* were tested in this study. Notably, a case of trans-differentiation from cervical mesonephric carcinoma to NEC was recently reported [82], which is thought to be the true primary HPV-negative NEC of the cervix. In this case, both mesonephric carcinoma and NEC components shared identical *U2AF1* and *GATA3* mutations and *MYCN* amplification. Furthermore, the NEC component harbored *TP53* and *MST1R* mutations that were not present in the mesonephric carcinoma. Together, these data suggest a clonal origin for the two components of this rare entity, rather than a collision tumor [82]. There have been no reports of cervical GAS or CCC showing neuroendocrine differentiation; however, such trans-differentiation to NEC might exist, as observed in primary gastric [132], pancreaticobiliary [133], and endometrial cancers [134]. When HPV-negative cervical NECs are identified, the possibility of cervical metastasis from more common NECs, such as lung or gastrointestinal primary tumors, should always be considered.

## 3. Animal Models of Cervical Cancer and Their Uses

Preclinical models of cervical cancer have greatly contributed to the understanding of cervical cancer carcinogenesis and tumor progression. To date, preclinical animal models of HPV-associated cervical squamous cell carcinoma have been developed and used for various research. These studies have revealed the process of tumorigenesis, interactions with the immune system, virus clearance, or establishment of latency [135,136]. Furthermore, viral tropisms and epithelial site-specific regulation of infection can be understood only through the use of animal models. For these purposes, various HPV16 transgenic models [137,138,139] have been utilized. Recent advances in animal models of cervical cancers shed light on carcinogenesis. He et al., established cervical epithelial cell-specific HPV16 E6/E7 and YAP1 double-knock in mouse model and revealed that the high-risk HPV synergized with hyperactivated YAP1 to promote the initiation and progression of cervical cancer [140]. Their findings indicated that hyperactivation of YAP1 in cervical epithelial cells facilitated HPV infection by increasing the putative HPV receptor molecules and disrupting host cell innate immunity [140]. Most recently, Henkle et al., developed a novel, spontaneous HPV16-expressing carcinoma model that captures major aspects of HPV-associated cervical cancer [141]. This mouse model showed expression of HPV E6/ E7 in the tumors of the female genital tract, spontaneous progression through HSIL to carcinoma, and flexibility to model cancers from different high-risk HPV genotypes. This model mouse was produced by injecting plasmids expressing HPV16 E6/E7-luciferase, AKT, c-myc, and Sleeping Beauty transposase into the cervicovaginal tract of C57BL/6 mice followed by electroporation. These tumor models may serve as important preclinical models for the development of therapeutic HPV vaccines or novel therapeutic interventions against HPV E6/E7-expressing tumors. 

Patient-derived xenograft (PDX) models is another valuable animal model which closely resemble the tumor features of patients and retain the molecular and histological features of diseases. A recent systematic review of PDX of cervical cancer revealed among 98 donor patients, 61 CC-PDX were established, and the overall success rate was 62.2% (0% to 75%) [142]. No correlation was found between the engraftment rate and characteristics of the tumor and donor patient, including histology, staging, and metastasis. However, a PDX model of HPV-negative cervical cancer has not been reported to date. Despite the utility of preclinical animal models, we need a novel animal model of HPV-negative uterine cervical cancer. To date, several animal models for HPV-negative squamous cell carcinoma of the head and neck or anal origin have been reported [143,144,145,146]. However, there is no animal model of HPV-negative uterine cervical squamous cell carcinoma. Furthermore, no preclinical model of HPV-negative cervical adenocarcinoma, such as gastric-type adenocarcinoma, has been developed. The development of animal models of these HPV-negative cervical cancers would reveal their carcinogenesis, and PDX models of them would contribute to future treatment development.

## 4. Future Directions and Conclusions

Although treatment for cervical cancer is not currently stratified based on HPV positivity [51,147], HPV-negative cervical cancers exist and have a worse prognosis than HPV-positive cervical cancers [148,149]. “HPV-negative cancers have reportedly shown worse survival than HPV-positive cervical cancers. 

Several studies have reported that women with HPV-negative tumors are more frequently diagnosed at advanced stages, with a higher rate of lymph node metastasis and impaired disease-free survival (DFS) and overall survival (OS) [57,58,60,148,149]. Rodriguez-Carunchio et al. reported that HPV-negative cervical cancer had a worse DFS than HPV-positive cancer (51.9 months, 95% CI (12.2–91.7) vs. 109.9 months, 95% CI (98.2–121.5), *p* = 0.01). Interestingly, the association between HPV status and DFS persisted even when adjusting for multiple covariates. No differences were observed in terms of DFS or OS after grouping patients according to histological type (SCCs vs. ADCs) [56]. In another study, Nicolas et al. reported that 21 (10%) out of 214 cervical cancers were negative for HPV DNA. HPV-negative cervical cancers frequently exhibited non-squamous histology compared with HPV-positive cervical cancers (9/21, 43% vs. 37/193, 19%; *p* < 0.01). HPV-negative cases were more frequently diagnosed at advanced stage (19/21, 91% vs. 110/193, 57%; *p* < 0.01) and more frequently had lymph node metastases (14/21, 67% vs. 69/193, 36%; *p* < 0.01). Patients with HPV-negative cervical cancer had a significantly shorter DFS (59.8 months, 95% CI 32.0–87.6 vs. 132.2 months, 95% CI 118.6–145.8; *p* < 0.01) and OS (77.0 months, 95% CI 47.2–106.8 vs. 153.8 months, 95% CI 142.0–165.6; *p* = 0.01) than patients with HPV-positive cancers [150]. However, a link between HPV-negativity and a poor clinical outcome has not yet been explained in terms of molecular abnormalities.

It is also becoming clear that HPV-negative cervical cancers have distinct clinicopathological and genetic features, and that their treatment could be tailored to different molecular therapeutic targets. Currently, cervical cancer treatment is not stratified according to HPV status or histology [151,152]. For invasive cervical cancer, the two standard curative treatment strategies are radical hysterectomy with pelvic and paraaortic lymphadenectomy and chemoradiation, which combines radiation therapy and concurrent platinum-based chemotherapy [151]. Furthermore, immunotherapy and molecular targeted therapy have advanced dramatically, and a wide range of treatments have been developed [153,154]. Recently, Colombo and the KEYNOTE-826 investigators confirmed the survival benefits of pembrolizumab in patients with persistent, recurrent, or metastatic cervical cancer, who were also receiving chemotherapy with or without bevacizumab in a randomized phase III trial [155]. Furthermore, therapeutic agents tailored according to molecular targets are being developed. These include drugs that target VEGF, EGFR, HER2, PI3K/AKT/mTOR, DNA damage repair, tissue factors, and other targets [153,154]. It is hoped that identifying these therapeutic targets will eventually lead to the selection of appropriate therapy, even for patients with rare HPV-negative cancers. Furthermore, a more comprehensive molecular analysis based on accurate pathological diagnoses may lead to the identification of new therapeutic targets for rare HPV-negative cancers.

However, false-negative HPV tests should also be considered, particularly those that occur because of improper specimen processing [34]. Furthermore, cervical metastasis from cancers originating from other organs should be ruled out as a possibility, as the treatment options differ greatly. As HPV vaccines have become more widely available, the number of deaths from HPV-positive cervical cancer is steadily declining [10]. However, HPV-negative cervical cancer is expected to persist in the post-vaccination era, necessitating intensive research into carcinogenic pathways and therapeutic targets.

## Figures and Tables

**Table 1:** Possible causes of false-negative HPV test results and non-cervical cancer misclassification.

Cause	Examples	Solution
**False-negative HPV test results**		
Deletion of targeted HPV DNA fragments during host genome integration	Cases with L1 loss during host genome integration can appear HPV-negative in tests covering only L1 fragments	Select a standard HPV testing method covering E6/E7 regions
Very low viral load in latent HPV infections	Less than 0.1% of HPV-negative cases develop HSIL or cervical cancer within 3–5 years	It may not be a problem for clinically evident cancers. Use p16 IHC and/or HPV ISH for tissue samples
Undetectable cervical cancer caused by non-high-risk HPV	Approximately 1–2% of cervical cancers are associated with non-high-risk HPV infection	Non-high-risk HPV may be a coincidental multiple infection or causal carcinogen. Combining different types of HPV tests may help
Inadequate sampling and various pre-analytical factors associated with HPV testing	False negatives are often the result of poor quality, insufficient tumor cells, or inadequate specimen fixation	Quality control of sampling methods, specimen handling, and processing to prevent HPV DNA degradation
**Non-cervical cancer misclassification**		
Cervical involvement of endometrial cancer	Endometrial endometrioid carcinoma with squamous differentiation misinterpreted as cervical adenosquamous carcinoma	IHC panel including p16, with HPV ISH for difficult cases
Metastasis to the uterine cervix	High-grade serous fallopian tube carcinoma implanted in the cervix or small cell lung carcinoma metastasized to the cervix	A careful review of clinical and radiological findings with IHC and/or HPV ISH

HPV, human papillomavirus; HSIL, high-grade squamous intraepithelial lesion; IHC, immunohistochemistry; ISH, in situ hybridization.

**Table 3:** 2020 WHO classification of cervical cancers and suggested non-invasive lesions.

Invasive Carcinoma	Non-Invasive Lesion
Squamous tumors	
SqCC, HPV-associated	High-grade squamous intraepithelial lesion
**SqCC, HPV-independent**	
SqCC NOS	
Glandular tumors	
Adenocarcinoma NOS	Adenocarcinoma in situ NOS
Adenocarcinoma, HPV-associated	Adenocarcinoma in situ, HPV-associated
Usual type, villoglandular, intestinal type, signet-ring, iSMILE	
**Adenocarcinoma, HPV-independent,** **gastric type**	**Adenocarcinoma in situ, HPV-independent: atypical LEGH, gastric type AIS**
**Adenocarcinoma, HPV-independent,** **clear cell type**	*Tubo-endometrioid metaplasia with atypia?* [53]
**Adenocarcinoma, HPV-independent,** **mesonephric type**	*Atypical mesonephric hyperplasia?* [54]
**Adenocarcinoma, HPV-independent, NOS**	
**Endometrioid adenocarcinoma NOS**	*Cervical endometriosis?* [55]
Carcinosarcoma NOS	
Adenosquamous carcinoma	
Mucoepidermoid carcinoma	
Adenoid basal carcinoma	
Carcinoma, undifferentiated, NOS	
Neuroendocrine tumors *	
Small/large cell neuroendocrine carcinoma	
Combined small/large cell neuroendocrine carcinoma	

SqCC, squamous cell carcinoma. * Neuroendocrine carcinomas in the female genital tract are described in a separate chapter. The suggested pre-invasive lesions depicted in italics are not established nor described in the 2020 WHO classification.

**Table 4:** Significantly mutated genes (SMGs) in whole exome/whole genome sequencing of cervical cancer.

Author/Year	Cases	SMGs
**Ojesina** **2014 [61]**	*n* = 115 (WES) HPV-positive (96%) SqCC (*n* = 79), ADC (*n* = 24), ADSC (*n* = 7), Others (N = 5)	SqCC: *PIK3CA* (14%), *EP300* (16%), *FBXW7* (15%), *PTEN* (6%), *STK11* (4%), *HLA-B* (9%), *MAPK1* (8%), *NFE2L2* (4%), *TP53* (9%), *ERBB2* (5%), ADC: *ELF3* (13%), *CBFB* (8%), *PIK3CA* (16%), *KRAS* (8%)
**TCGA** **2017 [22]**	*n* = 178 (core set, WES) HPV-positive (95%) SqCC (*n* = 144), ADC (*n* = 31), ADSC (*n* = 3)	*SHKBP1* (2%), *ERBB3* (6%), *CASP8* (4%), *HLA-A* (8%), *TGFBR2* (3%), *PIK3CA* (26%), *EP300* (11%), *FBXW7* (11%), *HLA-B* (6%), *PTEN* (8%), *NFE2L2* (7%), *ARID1A* (7%), *KRAS* (6%), *MAPK1* (5%)
**Huang** **2019 [63]**	*n* = 102 (WES) HPV-positive (93%) SqCC (*n* = 93), ADC (*n* = 6), ADSC (*n* = 3)	*PIK3CA* (16.7%), *FBXW7* (12.8%), *MLL3* (7.8%), *CASP8* (3.9%), *FADD* (3.9%)
**Zammataro** **2019 [65]**	*n* = 69 (WES) HPV-positive (100%) SqCC (*n* = 44), ADC (*n* = 17), ADSC (*n* = 6)	*PIK3CA* (27.5%), *STK11* (8.7%), *HUWE1* (15.9%), *FAT1* (15.9%), *NIPBL* (11.6%), *EPPK1* (10%), *BCORL1* (8.7%), *FBXW7, SMARCA4, NUP98, KRAS* (all 7.2%), *SMAD4* (5.8%), *MAPK1* (4.3%)
**Gagliardi** **2020 [66]**	*n* = 118 (WGS) HPV-positive (100%), SqCC (*n* = 97), ADC (*n* = 8), ADSC (*n* = 10), Others (*n* = 3)	*PIK3CA* (35%), *FAT1* (19%), *KMT2D* (14%), *FBXW7* (10%), *CASP8* (7%), *SLC35G5* (7%), *PCDHGA12* (6%), *MAPK1* (5%), *PSPC1* (5%), *ZNF750* (4%), *PCDHA9* (3%), *ZC3H6* (3%)

Abbreviations: SMGs, significantly mutated genes; WES, whole exome sequencing; HPV, human papilloma virus; SqCC, squamous cell carcinoma; ADC, adenocarcinoma; ADSC, adenosquamous carcinoma; WGS, whole genome sequencing; HIV, human immunodeficiency.

**Table 5:** Reported genetic alterations in HPV-negative cervical cancer of each histological type.

Histological Type	N	Method	SNVindel	Structural Variants	Ref
HPV-negative squamous cell carcinoma	4	Whole exome sequencing (WES)	*CHD8 (3/4), PIK3CA (2/4), LRP2 (2/4), COL7A1 (2/4), MTFR (2/4), PTEN (2/4)*	No recurrent CNV	[22]
Gastric-type adenocarcinoma	68	MSK-IMPACT 410–468 genes	*TP53 (28/68), CDKN2A (12/68), KRAS (12/68), STK11 (7/68), ERBB3 (7/68), GNAS (6/68), ERBB2 (6/68), SMAD4 (6/68), PIK3CA (5/68), ARID1A (4/68)*	No recurrent CNV	[74]
	21	Targeted sequencing 96 genes	*TP53 (11/21), STK11 (4/21), HLA-B (4/21), PTPRS (4/21), FGFR4 (3/21), GNAS (2/21), BRCA2 (2/21), ELF3 (2/21), ERBB3 (2/21), KMT2D (2/21), SLX4 (2/21)*	-	[75]
	15	YuanSu 450 panel 450 genes/39 fusions	*TP53 (8/15), STK11 (5/15), CDKN2A (4/15), ARID1A (3/15), PTEN (3/15)*	*ERBB2, CDK12, MECOM, PRKC1 (a// 2/15)*	[76]
	14	The Oncomine assay v3 161 genes	*TP53 (7/14) MSH6 (6/14), CDKN2A/B (5/14), POLE (5/14), SLX4 (5/14), ARID1A (4/14), STK11 (4/14), BRCA2 (3/14), MSH2 (3/14)*	*MDM2 (2/14)*	[77]
	11	Targeted sequencing 447 genes/60 fusions	*KRAS (4/11), TP53 (5/11), and PIK3CA (2/11), STK11 (3/11), CDKN2A (3/11), ATM (2/11), NTRK3 (2/11)*	No recurrent CNV	[78]
	3	Targeted sequencing 48 genes	*p53 (2/3), CDKN2A (2/3), KRAS (1/3), AKT1 (1/3), STK11 (1/3)*	-	[44]
Clear cell carcinoma	3	Targeted sequencing 48 genes	*TP53 (1/3), PIK3CA (1/3)*	-	[44]
	1	Targeted sequencing 447 genes/60 fusions	*POLE P286R and 201 additional mutations, including in PIK3CA, ARID1A, and PTEN.*	Not detected	[79]
Mesonephric carcinoma	13	Targeted sequencing 413 genes/35 fusions	*KRAS (9/13), ARID1A/B (7/13), BCOR/BCOL1 (5/13), SMARCA4 (2/13)*	Gain of 1q (9/13)	[80]
	4	OncomineComprehensive assay v1 143 genes/22 fusions	*KRAS (4/4), PIK3CA (1/4)*	Gain of 1q (4/4)	[54]
	1	FoundationOne CDx	*KRAS, CTNNB1*	Gain of 1q	[81]
Endometrioid carcinoma	8	Targeted sequencing 48 genes	*PIK3CA (4/8), PTEN (4/8), CTNNB1 (3/8), FBXW7 (2/8), KRAS (1/8), AKT1 (1/8), TP53(1/8), MSI-H (1/8)*	-	[44]
Serous carcinomoa	6	Targeted sequencing 48 genes	*TP53 (4/6), KRAS (2/6), PIK3CA (1/6), PTEN (1/6)*	-	[44]
Adenosquamous carcinoma	3	Targeted sequencing 50 genes	Not detected	-	[49]
	1	WES	Not detected	Not detected	[22]
Neuroendocrine carcinoma	14	Foundation Medicine 315 genes/19 fusions	*PTEN (7/14), TP53 (6/14), PIK3CA (5/14), ARID1A (5/14), RB1 (5/14)*	*MYC (1/14)*	[48]
Mixed mesonephric adenocarcinoma and neuroendocrine carcinoma	1	MSK-IMPACT 410–468 genes	*U2AF1, GATA3, TP53, MST1R*	*MYCN, ISR2*	[82]

Abbreviations: SNVindel, single nucleotide variations, insertions/deletions; CNV, copy number variation.
